# Research trends and hotspots on the links between caveolin and cancer: bibliometric and visual analysis from 2003 to 2022

**DOI:** 10.3389/fphar.2023.1237456

**Published:** 2023-07-28

**Authors:** Yaqian Tan, Qi Song

**Affiliations:** ^1^ Department of Pharmacy, The Affiliated Brain Hospital of Guangzhou Medical University, Guangzhou, China; ^2^ Department of Pharmacy, Affiliated Cancer Hospital and Institute of Guangzhou Medical University, Guangzhou, China

**Keywords:** bibliometric analysis, visualization, caveolin, cancer, VOSviewer, CiteSpace, R-bibliometrix

## Abstract

**Introduction:** Extensive studies indicated that caveolin is a key regulator in multiple cellular processes. Recently, growing evidence demonstrated that caveolin is critically involved in tumor progression. Since no relevant bibliometric study has been published, we performed a bibliometric and visual analysis to depict the knowledge framework of research related to the involvement of caveolin in cancer.

**Methods:** Relevant studies published in English during 2003–2022 were obtained from the Web of Science Core Collection database. Three programs (VOSviewer, CiteSpace, and R-*bibliometrix*) and the website of bibliometrics (http://bibliometric.com/) were applied to construct networks based on the analysis of countries, institutions, authors, journals, references, and keywords.

**Results:** A total of 2,463 documents were extracted and identified. The United States had the greatest number of publications and total citations, and Thomas Jefferson University was the most productive institution. Michael P. Lisanti was the most influential scholar in this research domain. *Cell Cycle* was the journal with the most publications on this subject. The most local-cited document was the article titled “Caveolin-1 in oncogenic transformation, cancer, and metastasis.” A comprehensive analysis has been conducted based on keywords and cited references. Initially, the research frontiers were predominantly “signal transduction”, “human breast cancer,” “oncogenically transformed cells,” “tumor suppressor gene,” and “fibroblasts.” While in recent years, the research emphasis has shifted to “tumor microenvironment,” “epithelial mesenchymal transition,” “nanoparticles,” and “stem cells.”

**Conclusion:** Taken together, our bibliometric analysis shows that caveolin continues to be of interest in cancer research. The hotspots and research frontiers have evolved from the regulation of cancer signaling, to potential targets of cancer therapy and novel techniques. These results can provide a data-based reference for the guidance of future research.

## Introduction

Cancer is a leading cause of death in the world, and malignant tumors were responsible for approximately 7 million deaths annually (Torre et al., 2016). The global burden of cancer has been further increased due to population aging as well as lifestyles that are known to increase cancer risk, such as alcohol consumption, smoking, poor diet, and obesity (Yang et al., 2018). It is relatively rare for cancer to develop during the average human lifespan because of our intrinsic anti-cancer mechanisms. However, cancer cells can evade defense mechanisms by developing capabilities such as angiogenesis induction, insensitivity to anti-growth signals, drug resistance, and apoptosis resistance (Hanahan and Weinberg, 2000).

Caveolin is a multifunctional scaffolding protein of caveolae, and evidence suggested that caveolin is a key regulator in multiple cellular processes (Williams and Lisanti, 2004; Williams et al., 2005; Volonte and Galbiati, 2020). As a protein family, caveolin has three members: caveolin-1, caveolin-2, and caveolin-3. Caveolin-1 and caveolin-2 are found in all types of cells, and caveolin-3 is mostly expressed in skeletal muscles (Liu et al., 2002; Gupta et al., 2014). Caveolin has been reported to influence various capabilities of cancer cells, supporting the idea that caveolin is a key regulator in the development of cancer (Volonte and Galbiati, 2020). Growing evidence further demonstrated that caveolin contributes significantly in the regulation of multiple cellular processes of cancer, including migration, metastasis, survival, and angiogenesis (Goetz et al., 2008; Ho et al., 2008; Patani et al., 2012; Tang et al., 2012; Faggi et al., 2015; Maiuthed et al., 2018; Yamao et al., 2019).

Since studies related to the involvement of caveolin in cancer have developed rapidly, it is necessary to systematically explore the current status and future trends on this subject. Bibliometrics is a common method for depicting the knowledge structure and frontiers of a specific field (Chen and Song, 2019; Xu et al., 2022). Although many review articles have examined the relationship between caveolin and cancer, bibliometric analysis has not, as yet, been seen in this field. Therefore, in the current study, we conducted a bibliometric and visual analysis to summarize the publications over the past 2 decades. We hope that this study can build a comprehensive picture of research on the links between caveolin and cancer, and provide an effective reference for scholars to better investigate the history and future directions in this research domain.

## Materials and methods

### Data acquisition

**FIGURE 1:**
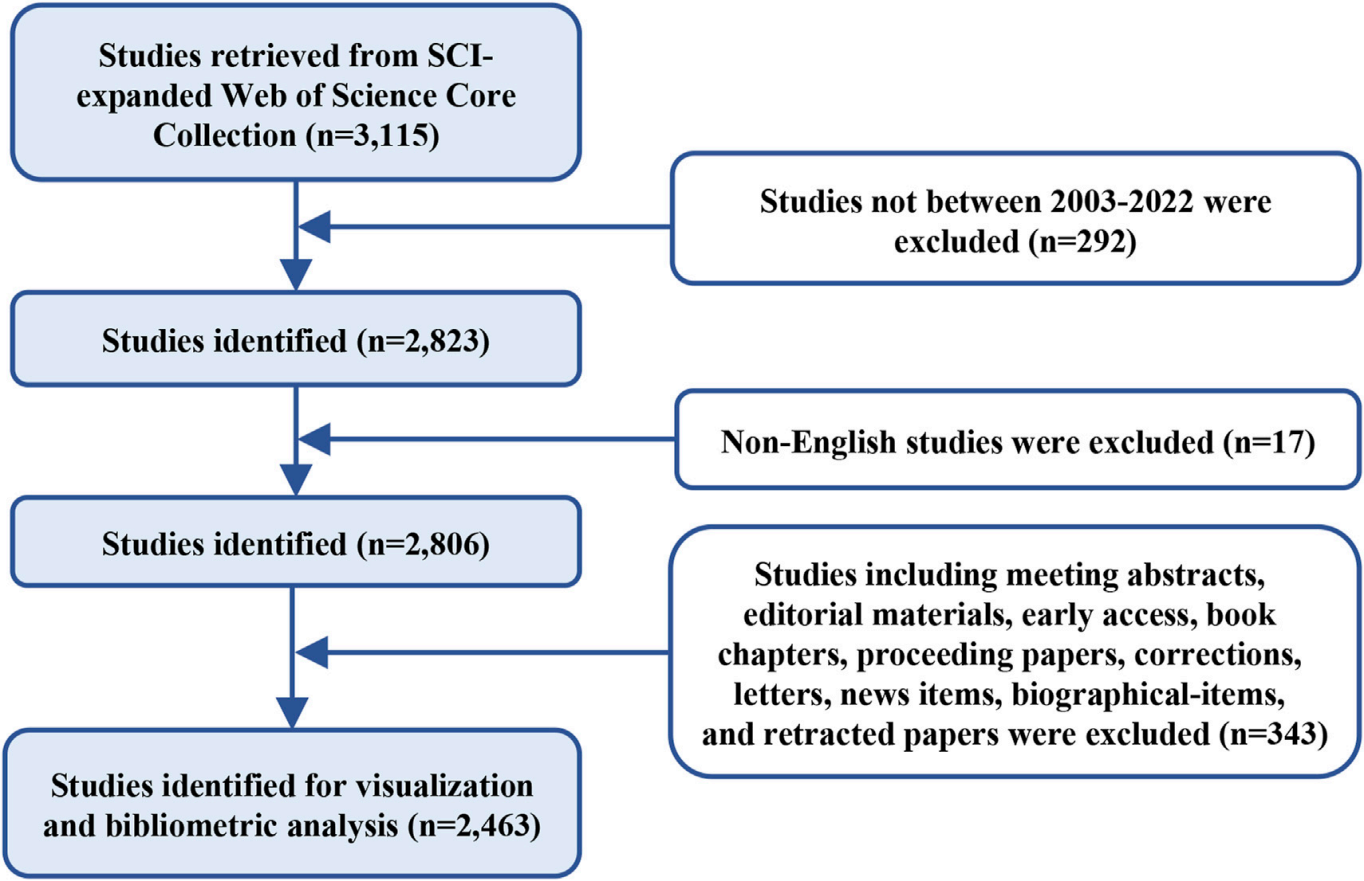
Flowchart of data collection.

Data retrieval was performed on 22 April 2023 using the Web of Science Core Collection (WOSCC) database, and the search formula was set to TS = (cancer* OR carcinoma* OR neoplasms* OR sarcoma* OR tumor* OR tumour* OR lymphoma* OR leukemia* OR leukaemia* OR malignan*) AND TS = (caveolin*). The time frame was set to 2003–2022 encompassing 20 years, and non-English studies were excluded. Only articles and reviews were included and irrelevant documents (meeting abstracts, biographical-items, editorial materials, early access, letters, book chapters, proceeding papers, corrections, news items, and retracted papers) were excluded (Figure 1).

### Data analysis

In our current study, VOSviewer, CiteSpace, R–*bibliometrix*, and the website of bibliometrics (http://bibliometric.com/), were applied to perform bibliometric and visual analysis.

VOSviewer (version 1.6.18) is a JAVA-based software invented by Professor Nees Jan van Eck and Professor Ludo Waltman (Leiden University, the Netherlands) (van Eck and Waltman, 2010). This software can be applied to analyze the network of countries, institutions, authors, journals, and keywords based on collaborative data or co-occurrence data. In this study, VOSviewer was employed to visualize the institution co-authorship network and keywords co-occurrence map.

CiteSpace (version 6.2.R2) is also a JAVA-based software developed by Professor Chaomei Chen (Drexel University, the United States). It can be employed to combine bibliometric analysis and data mining algorithms for the final visualization (Chen, 2004). In this study, CiteSpace was used to visualize the clusters of cited references by using the LLR (log-likelihood ratio) algorithm. In addition, burst analysis of keywords was applied as an indicator of the research emphasis.

The R–*bibliometrix* (version 4.1.0) is an R-based package built by Professor Massimo Aria (University of Naples Federico II, Italy) and Professor Corrado Cuccurullo (University of Campania Luigi Vanvitelli, Italy). In the current study, R–*bibliometrix* was employed to conduct a comprehensive investigation of the countries, institutions, authors, journals, references, and keywords (Aria and Cuccurullo, 2017). The top journals were determined using the Bradford’s law (Brookes, 1985), whereas the *h*-index and *g*-index were applied to measure the academic impact of authors (Hirsch, 2005; Ali, 2021).

The website of bibliometrics (http://bibliometric.com/) was applied to present the global cooperation atlas.

## Results

### Analysis of general trend

**FIGURE 2:**
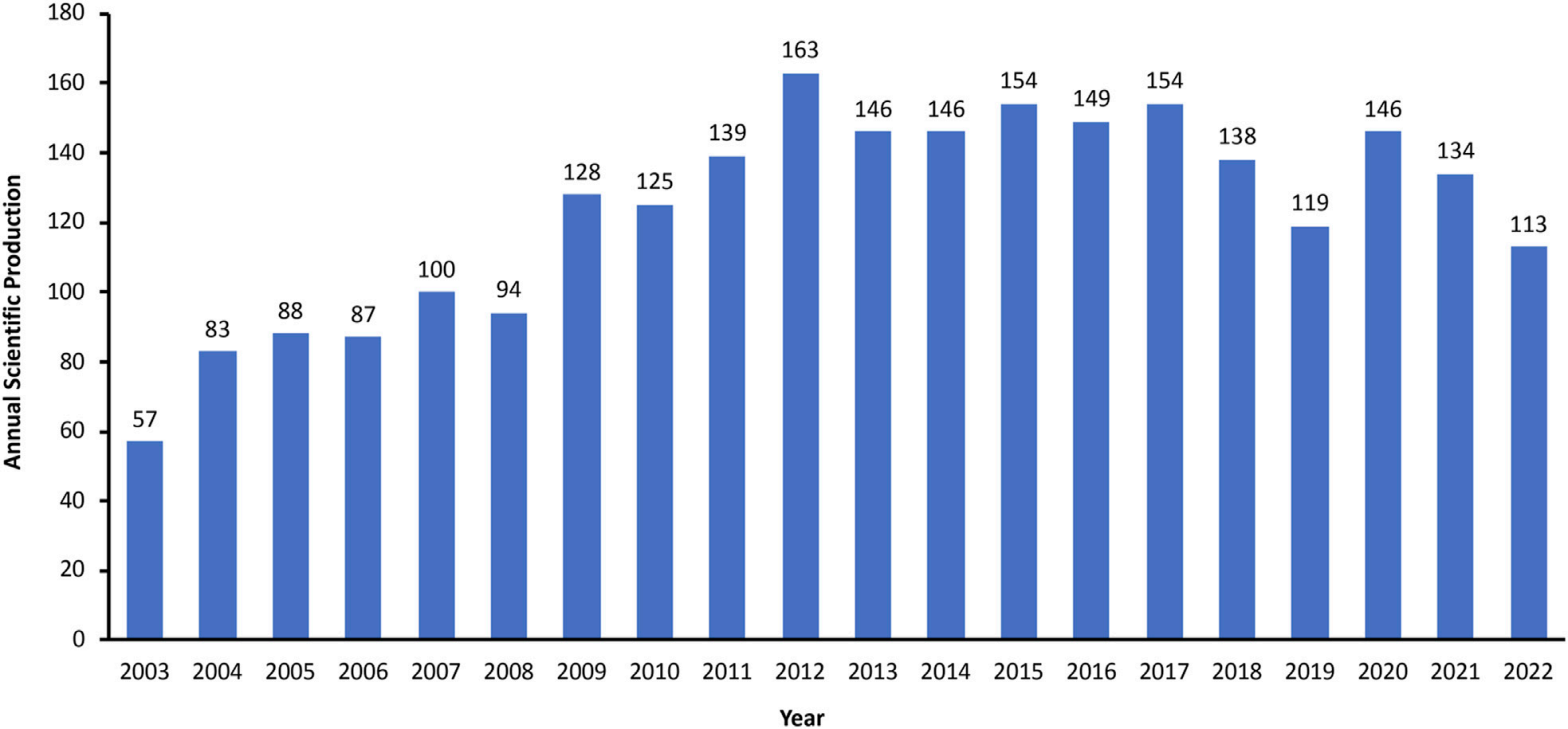
Annual global output between 2003 and 2022.

In this study, a total of 2,463 related documents were identified and met the inclusion criteria, and the annual scientific productions showed a general ascending trend (Figure 2). Prior to 2009, research with regard to the links between caveolin and cancer was relatively slow to develop, and none of them had more than 100 annual publications. The annual productions began to increase in 2009 and reached a peak of 163 publications in 2012. After 2012, the number of annual publications stayed relatively stable, fluctuating around 150 for the majority of the period.

### Analysis of countries

**FIGURE 3:**
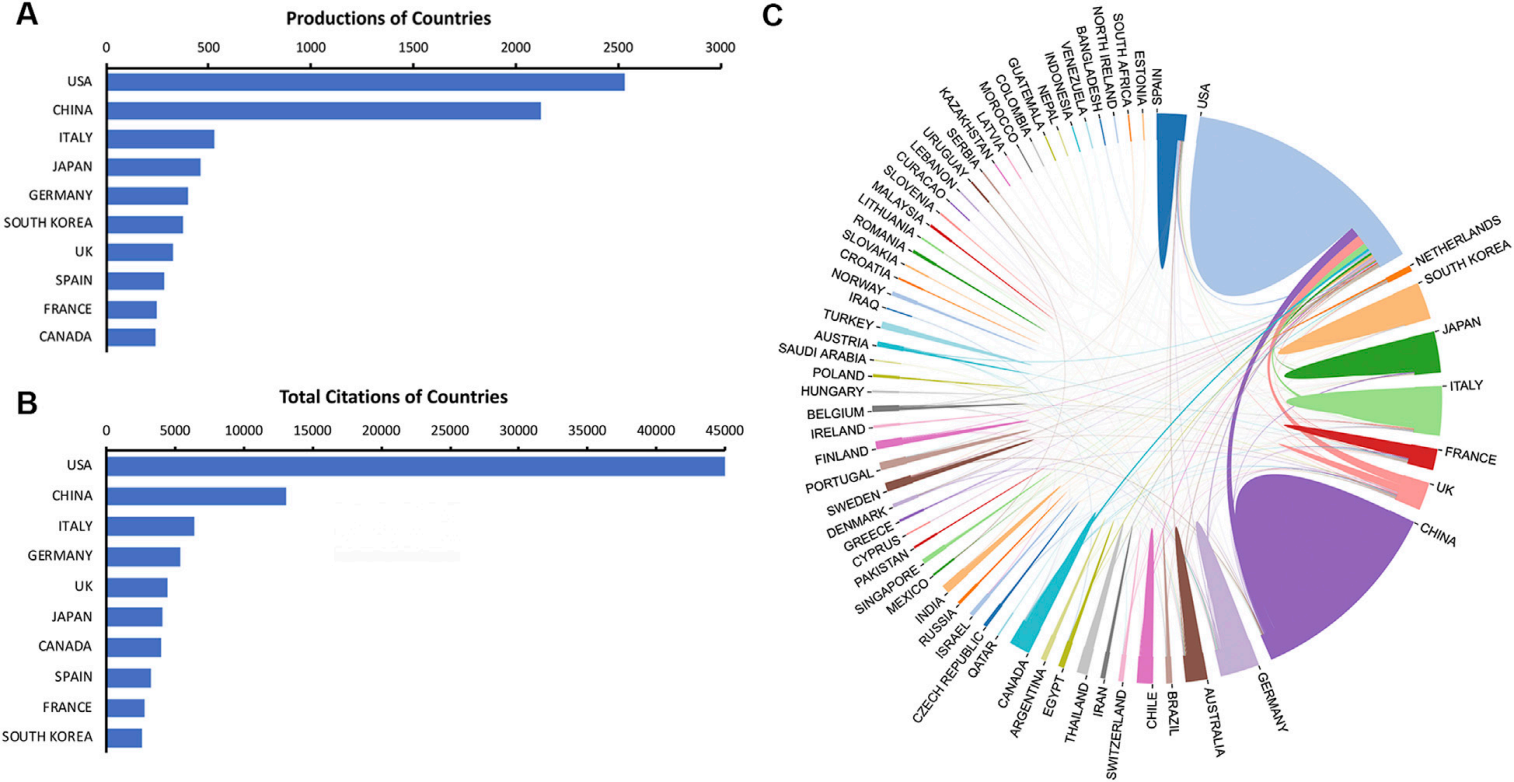
Contributions of countries. **(A)** The top ten countries in publications. **(B)** The top ten countries in total citations. **(C)** Cooperation networks of active countries.

A total of 65 countries have contributed relevant literature. As shown in Figure 3A, the United States ranked first in terms of publications (2,534 publications, 27.39%), followed by China (2,125 publications, 22.97%) and Italy (529 publications, 5.72%). These three countries contributed approximately 56.08% of total publications in this field from 2003 to 2022. Figure 3B exhibits that the United States, China, and Italy also ranked the top three with regard to the total citations (United States: 44,993 citations, China:130,49 citations, Italy: 6,350 citations). A total of 371 collaborations were identified in the global cooperation networks, and the top three were USA-China (90 collaborations), USA-UK (89 collaborations), and USA-Italy (58 collaborations) (Figure 3C).

### Analysis of institutions

**FIGURE 4:**
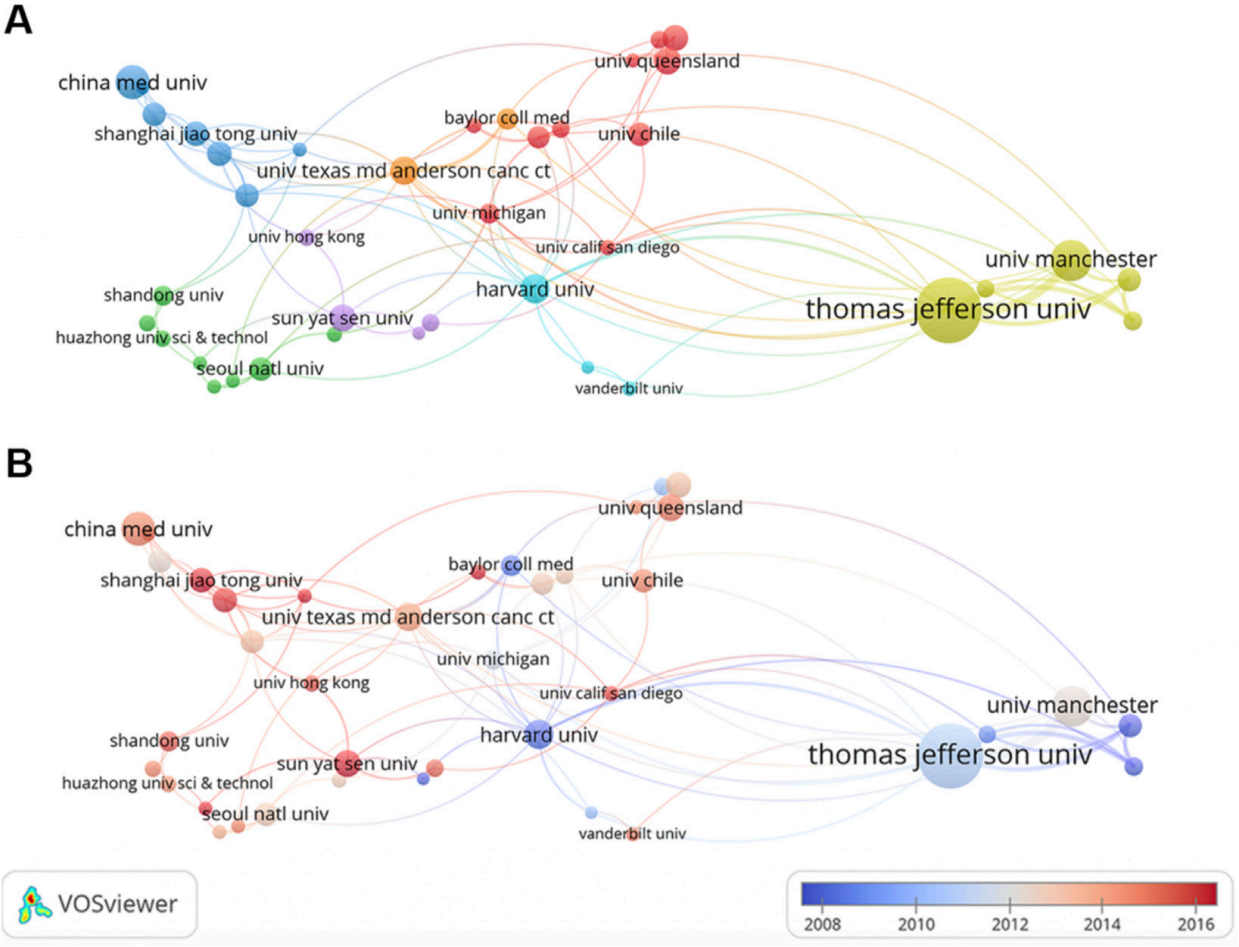
Co-authorship analysis of institutions. **(A)** Cluster visualization for institution co-authorship network. **(B)** Timeline visualization for institution co-authorship network.

During 2003–2022, a total of 2,438 institutions launched papers on this theme. As listed in Table 1, the top three institutions were Thomas Jefferson University (372 publications), University of Manchester (115 publications), and China Medical University (101 publications). Institutions with 15 or more publications were included in the collaboration network analysis and then visualized by VOSviewer. The clusters are organized in different colors by the frequency of cooperation between each institution (Figure 4A). Thomas Jefferson University was represented by the largest node (with a total link strength of 90), indicating the highest degree of cooperation with other institutions. The strongest link was between Thomas Jefferson University and University of Manchester (with a link strength of 51), and they were thus connected by the thickest line. Figure 4B depicts the timeline visualization for institution co-authorship network. The average publication year of an institution was represented by the color of the node according to the chronological gradient. Harvard University (with an average publication year of 2008.35) was shown in dark blue, indicating that researchers from Harvard University were active during the earlier stage of this research field. Shanghai Jiao Tong University (with an average publication year of 2017.18) was shown in dark red, indicating that this university was more recently active in this field.

**TABLE 1:** The top ten most active institutions.

Rank	Institution	Country	Publications	Total citations	Average citations
1	Thomas Jefferson University	United States	372	10,914	29.34
2	University of Manchester	United Kingdom	115	3,104	26.99
3	China Medical University	China	101	457	4.52
4	University of Texas MD Anderson Cancer Center	United States	93	654	7.03
5	Chulalongkorn University	Thailand	92	523	5.68
6	University of Queensland	Australia	87	1,333	15.32
7	Albert Einstein College of Medicine	United States	76	2,451	32.25
8	University of Chile	Chile	71	1,070	15.07
9	Baylor College of Medicine	United States	67	1,657	24.73
10	Fudan University	China	67	363	5.42

### Analysis of authors

**FIGURE 5:**
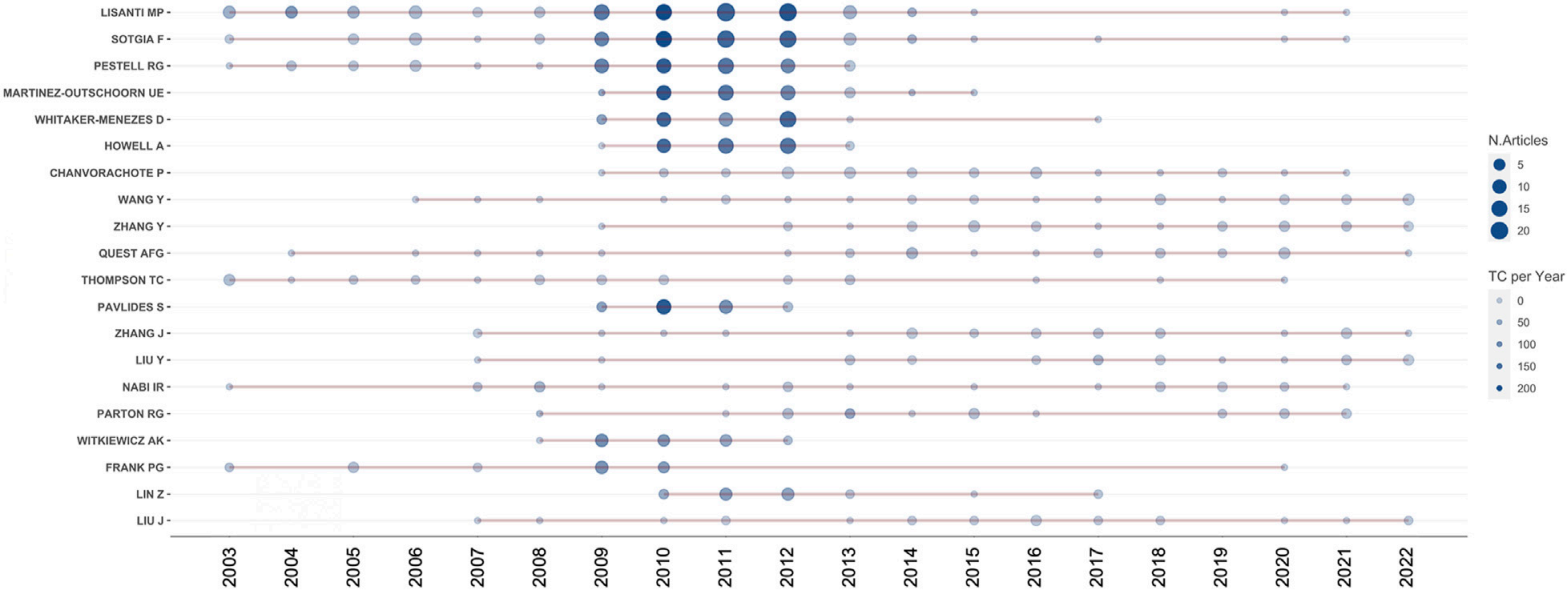
Timeline visualization for the top twenty active researchers. The number of publications and citations are represented by the size and color of the node, respectively.

A total of 12,168 authors published relevant papers, and the top ten most fruitful authors are listed in Table 2. Michael P. Lisanti (University of Salford, United Kingdom) was the most influential author with 119 publications and 14,698 total citations, followed by Federica Sotgia (University of Salford, United Kingdom) and Richard G. Pestell (Thomas Jefferson University, United States), whereas the remaining authors had fewer than 50 publications. Michael P. Lisanti, Federica Sotgia, and Richard G. Pestell also ranked the top three authors according to the *h*-index and *g*-index values. Figure 5 shows the timeline visualization for the top twenty active researchers and their productions over time.

**TABLE 2:** The top ten most productive authors.

Rank	Author	Institution	Publications	Total citations	h-index	g-index	
1	Michael P. Lisanti	University of Salford	119	14,698	72	119	
2	Federica Sotgia	University of Salford	93	10,598	62	93	
3	Richard G. Pestell	Thomas Jefferson University	67	8,693	53	67	
4	Ubaldo E. Martinez-Outschoorn	Thomas Jefferson University	45	7,209	42	45	
5	Diana Whitaker-Menezes	Thomas Jefferson University	43	6,347	39	43	
6	Anthony Howell	Thomas Jefferson University	42	5,679	38	42	
7	Pithi Chanvorachote	Chulalongkorn University	33	929	18	30	
8	Ying Zhang	Harbin Medical University	30	539	15	22	
9	Yu Wang	Sichuan University	30	499	13	22	
10	Timothy C. Thompson	University of Texas MD Anderson Cancer Center	28	1,536	21	28	

### Analysis of journals

The top ten most relevant journals are listed in Table 3, *Cell Cycle* (68 publications) was the journal with the most prolific outlet, followed by *Journal of Biological Chemistry* (67 publications) and *PLoS One* (65 publications). According to the Journal Citation Reports (JCR) 2022, five journals were located in Q1 region, and four journals were located in Q2 region. The impact factor (IF) ranged from 2.435 in *Anticancer Research* to 13.312 in *Cancer Research* among the top ten most relevant journals.

**TABLE 3:** The top ten most relevant journals.

Rank	Journal	Publications	Total citations	Average citations	If (2022)	JCR region
1	Cell Cycle	68	1,502	22.09	5.173	Q2
2	Journal of Biological Chemistry	67	775	11.57	5.486	Q2
3	PLoS One	65	343	5.28	3.752	Q2
4	Cancer Research	50	935	18.70	13.312	Q1
5	Oncogene	43	483	11.23	8.756	Q1
6	Scientific Reports	36	101	2.81	4.996	Q1
7	American Journal of Pathology	33	827	25.06	5.770	Q1
8	Biochemical and Biophysical Research Communications	31	91	2.94	3.322	Q2
9	Anticancer Research	30	233	7.77	2.435	Q4
10	Cancer Letters	29	188	6.48	9.756	Q1

### Analysis of references

There were two concepts in R-*bibliometrix*, “local citation” and “global citation”. Local citation represents the citations of a study in the similar fields, while global citation indicates the citations of a study in all fields. A higher local citation value reflects a higher degree of recognition in the peer field (Luo et al., 2022). As listed in Table 4, the literature with the highest value of local citation was the study published in *American Journal of Physiology-Cell Physiology* titled “Caveolin-1 in oncogenic transformation, cancer, and metastasis” (276 local citations), while the study with the greatest value of global citation was the paper published in *Physiological Reviews* titled “Role of caveolae and caveolins in health and disease” (691 global citations).

**TABLE 4:** The top ten highly cited documents.

Rank	Publication	DOI	Local citations	Global citations
1	Williams TM, 2005, *American Journal of Physiology-Cell Physiology*	10.1152/ajpcell.00458.2004	276	439
2	Goetz JG, 2008, *Cancer and Metastasis Reviews*	10.1007/s10555-008-9160-9	157	239
3	Witkiewicz AK, 2009, *American Journal of Pathology*	10.2353/ajpath. 2009.080873	149	252
4	Williams TM, 2004, *Journal of Biological Chemistry*	10.1074/jbc.M409214200	142	253
5	Cohen AW, 2004, *Physiological Reviews*	10.1152/physrev.00046.2003	140	691
6	Li LK, 2003, *Molecular and Cellular Biology*	10.1128/MCB.23.24.9389–9404.2003	124	259
7	Savage K, 2007, *Clinical Cancer Research*	10.1158/1078–0432.CCR-06–1371	104	181
8	Sloan EK, 2009, *American Journal of Pathology*	10.2353/ajpath. 2009.080924	102	165
9	Sunaga N, 2004, *Cancer Research*	10.1158/0008–5472.CAN-03–3941	101	156
10	Hill MM, 2008, *Cell*	10.1016/j.cell. 2007.11.042	100	517

**FIGURE 6:**
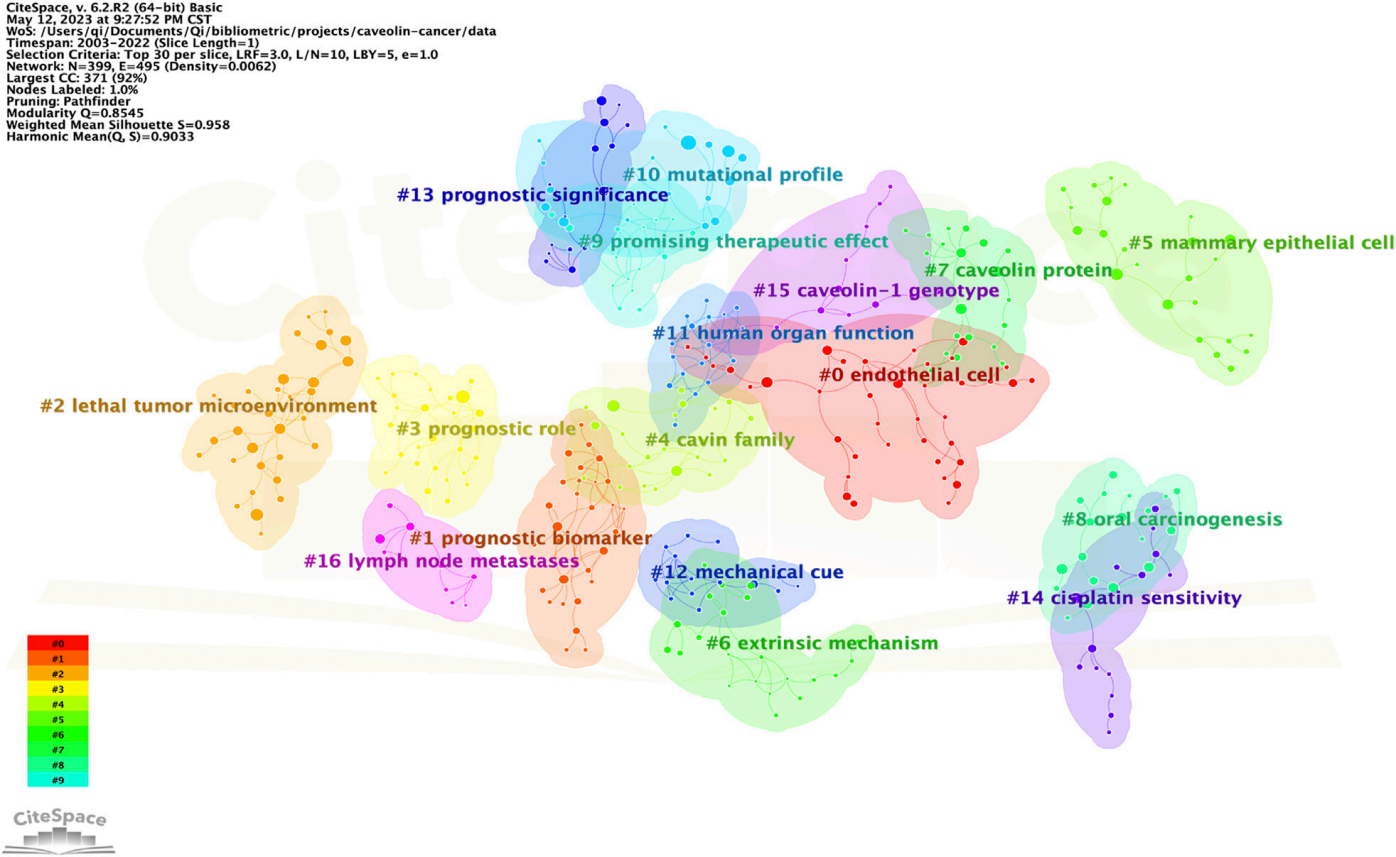
Cluster visualization for cited references.

The cluster visualization for cited references was conducted by CiteSpace, and a total of 17 clusters were formed according to the keywords of cited references (Figure 6). The labels of 17 clusters were: #0 endothelial cell, #1 prognosis biomarker, #2 lethal tumor microenvironment, #3 prognostic role, #4 cavin family, #5 mammary endothelial cell, #6 extrinsic mechanism, #7 caveolin protein, #8 oral carcinogenesis, #9 promising therapeutic effect, #10 mutational profile, #11 human organ function, #12 mechanical cue, #13 prognostic significance, #14 cisplatin sensitivity, #15 caveolin-1 genotype, #16 lymph node metastases.

### Analysis of keywords

Keywords analysis was conducted by VOSviewer and CiteSpace. The threshold of keywords occurrence was set to 10 considering the readability of the graph, and 446 keywords were included in the analysis.

**FIGURE 7:**
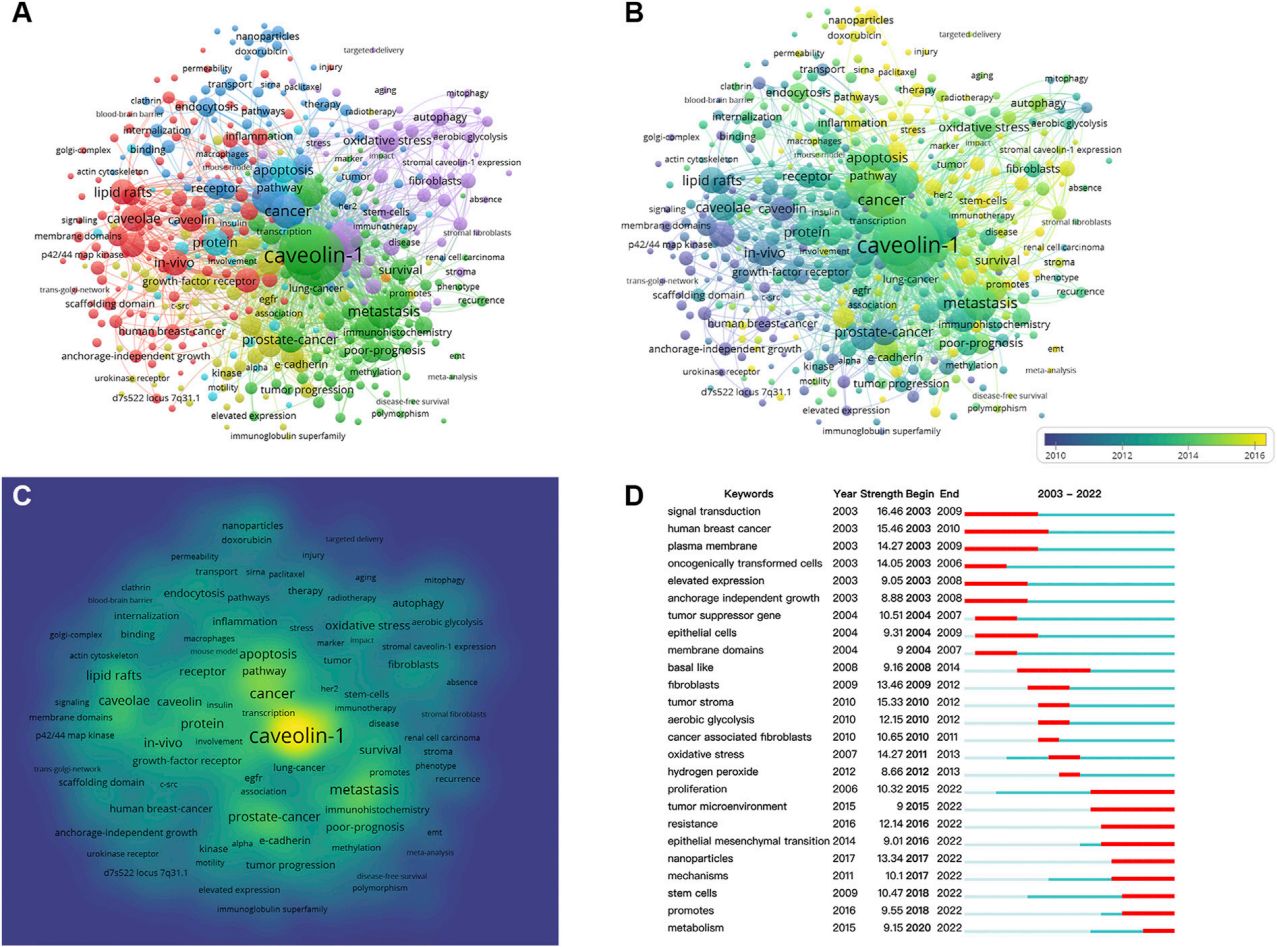
Co-occurrence analysis of keywords. **(A)** Cluster visualization for keywords. **(B)** Timeline visualization for keywords. **(C)** Frequency visualization for keywords. **(D)** Citation bursts of the top twenty-five keywords.

In Figure 7A, keywords were organized into 6 clusters labeled with different colors. In cluster 1, “caveolae” was the largest node, the other main nodes included lipid rafts, caveolin, gene expression, plasma-membrane, *in-vivo*, nitric oxide synthase, angiogenesis, inflammation, and signal-transduction. This cluster mainly focused on the structure of caveolin and the underlying mechanisms of cancer signaling. In cluster 2, “caveolin-1” was the largest node, the other main nodes included metastasis, progression, survival, overexpression, prostate-cancer, and adenocarcinoma. This cluster mainly focused on the associations between caveolin-1 overexpression and tumor progression. In cluster 3, “cancer” was the largest node, the other main nodes included activation, receptor, *in-vitro*, endocytosis, mechanisms, nanoparticles, and therapy. This cluster represented the potential targets and mechanisms for the therapeutics of cancer. In cluster 4, “downregulation” was the largest node, the other main nodes included upregulation, migration, invasion, and tyrosine phosphorylation. This cluster indicated that the regulation of caveolin on tyrosine phosphorylation was widely concerned. In cluster 5, “growth” was the largest node, the other main nodes included oxidative stress, autophagy, stem cells, and fibroblasts. This cluster represented the current research hotspots in cancer therapy. In cluster 6, “protein” was the largest node, the other main nodes included apoptosis, proliferation, and pathway. This cluster focused on proteins in different pathways of cell apoptosis and proliferation.

Figure 7B illustrates the timeline visualization for keywords, and the average occurrence time was represented by the color of the node according to the chronological gradient. The keyword “oncogenically transformed-cell” (with an average occurrence time of 2006.98) was shown in blue, revealing an early-stage research emphasis. The keywords “micelles” (with an average occurrence time of 2019.08) was shown in yellow, indicating that this keyword was more recently active in this field.

Figure 7C shows the heatmap of keywords frequency in which the yellow area represents a high frequency of occurrence. The most frequent keyword was “caveolin-1,” other hotspots included “metastasis,” “prostate-cancer,” “apoptosis,” “caveolae” and “lipid rafts.”

Figure 7D presents the citation bursts of the top twenty-five keywords, in which the time interval and duration of the citation burst were marked in blue and red, respectively. It is noticeable that keywords were divided into three stages by the duration of the citation burst. In the early stage (2003–2010), keywords included “signal transduction,” “human breast cancer,” “plasma membrane,” “oncogenically transformed cells,” “elevated expression,” “anchorage independent growth,” “tumor suppressor gene,” “epithelial cells,” and “membrane domains.” Research hotspots during 2008–2014 shifted to “basal like,” “fibroblasts,” “tumor stroma,” “aerobic glycolysis,” “oxidative stress,” and “hydrogen peroxide.” From 2015 to 2022, the main research frontiers were “proliferation,” “tumor microenvironment,” “resistance,” “epithelial mesenchymal transition,” “nanoparticles,” “mechanisms,” “stem cells,” “promotes,” and “metabolism.”

## Discussion

### General trends and knowledge structure of global publications

The development of a research topic can be reflected by the amount of annual scientific output (Durieux and Gevenois, 2010). As can be observed from the results, the annual publication number in this field showed a fluctuating but generally ascending trend prior to 2013 (with a growth rate of 12.38%), followed by a slightly descending trend during 2013–2022 (with a growth rate of −2.81%). This suggested that this topic had received significant attention in the first decade but entered a period of slow development from 2013, which might indicate that some challenges and bottlenecks appeared in some aspects of this research field and therefore innovation is required in the future investigation.

Our results of the national contributions suggested that the United States had great influence in this field by the leading numbers of publications and total citations. In addition, the United States constituted 33.70% of the amount of publications and 49.50% of the amount of total citations among the top ten most productive countries. Interestingly, although China ranked second in terms of both publications and total citations, we found that the number of publications from China (2,125 publications) was 83.86% of that from the United States (2,534 publications), whereas the number of total citations from China (13,049 citations) was only 29.00% of that from the United States (44,993 citations). This might reflect the global structure that the United States had deeper accumulation and more high-quality publications than other countries. According to our data on the institution distribution, the United States contributed four of the top ten institutions. Moreover, Thomas Jefferson University from the United States was the only institution with more than 300 publications and over 10,000 total citations, indicating the great impact and significant academic accumulation in this domain. Therefore, based on the contributions of countries and institutions, we can assume that the United States is having an absolute leading position in this field.

The *h*-index and *g*-index are generally applied to evaluate the academic impact of a researcher (Hirsch, 2005; Ali, 2021). From the perspective of authors, Michael P. Lisanti and Federica Sotgia, both from University of Salford in the United Kingdom, had significant impact in this field by their most prolific output, as well as their high values of *h*-index and *g*-index. In particular, Michael P. Lisanti undertook significant research in this field and formed close collaborations with researchers worldwide. As listed in Table 2, the following four active authors, Richard G. Pestell, Ubaldo E. Martinez-Outschoorn, Diana Whitaker-Menezes, and Anthony Howell, were all from Thomas Jefferson University in the United States, and the last four authors on the list were one scholar from Thailand and three scholars from China. This might reflect the late start of Thailand and China in this research field and the last four researchers on the list were therefore not as fruitful as the top six researchers.

Among the top ten most relevant journals, *Cell Cycle* had the most publications (68 publications), followed by *Journal of Biological Chemistry* (67 publications) and *PLoS One* (65 publications). As shown in Table 3, nine of the core journals were located in JCR region Q1 or Q2, and *Cancer Research* ranked first with the highest IF of 13.312. On the other hand, the top three most cited journals, *Cell Cycle* (1,502 total citations), *Cancer Research* (935 total citations), and *American Journal of Pathology* (827 total citations) and their high IF values (with IF values of 5.173, 13.312, and 5.770, respectively) revealed the strong influence and high quality in this field. Thus, these results will help scholars searching the core journals related to the links between caveolin and cancer.

The research frontiers of a topic can be reflected by the analysis of cited references. Local citation is indicative of the citations of a study in the similar fields, while global citation indicates the citations of a study in all fields. A higher local citation of the study reflects a higher degree of recognition in the peer field (Luo et al., 2022). The literature ranked first in local citation was the study published in *American Journal of Physiology-Cell Physiology* titled “Caveolin-1 in oncogenic transformation, cancer, and metastasis” (276 local citations), while the study published in *Physiological Reviews* titled “Role of caveolae and caveolins in health and disease” had the highest number of global citation (691 global citations). These highly cited publications are considered essential references for scholars working in this field. For example, Hill et al. explored the important role of caveolin formation (Hill et al., 2008), Sunaga et al. introduced the opposite effects of caveolin-1 on the progression of non-small cell lung cancer and small cell lung cancer (Sunaga et al., 2004), and Williams et al. summarized the functions of caveolin-1 in cancer migration and metastasis (Williams and Lisanti, 2005). Interestingly, after a comprehensive analysis of authors and cited references, we found that Michael P. Lisanti, as the corresponding author, appeared in four of the top ten highly cited publications. These findings further suggested that he is an accomplished researcher in this field.

### Research hotspots of global publications

In bibliometric analysis, keywords co-occurrence analysis can reflect core contents of the literature, and timeline visualization for keywords can reveal the evolution of research frontiers (Liu et al., 2014). Thus, we formed the evolution of keywords into three stages according to the citation bursts of keywords. In the early stage (2003–2010), keywords such as “signal transduction,” “human breast cancer,” “plasma membrane,” “oncogenically transformed cells,” “elevated expression,” “anchorage independent growth,” “tumor suppressor gene,” “epithelial cells,” and “membrane domains,” were mainly associated with cancer-related processes. In the following period (2008–2014), most terms focused on potential targets to the therapeutic of cancer, such as “fibroblasts,” “tumor stroma,” “aerobic glycolysis,” “oxidative stress,” and “hydrogen peroxide.” In recent years (2015–2022), “tumor microenvironment,” “epithelial mesenchymal transition,” “nanoparticles,” “stem cells,” and “metabolism” became the main research frontiers, and these keywords mainly focused on novel technologies and new directions. Interestingly, these data were in line with the timeline visualization (Figure 7B) and frequency visualization of keywords (Figure 7C) that the early-stage emphasis was mainly associated with mechanisms for cancer signaling, then the research hotspots shifted to the application of novel technologies in cancer therapy.

According to the cluster visualization of keywords and cited references, the evolution of studies on the links between caveolin and cancer could be summarized into the following three branches.

1) The general function of caveolin

Caveolae are flask-shaped invagination on the plasma membrane which was initially considered to be a simple membrane structure but later believed to be a more complex organelle related to cellular functions (Parton, 2018). Early in 1955, Yamada first observed caveolae as invaginations of the plasma membrane using electron microscopy (Yamada, 1955). Palade and Bruns subsequently identified caveolae in vascular endothelial cells and suggested its function of delivering molecules across endothelial cells (Palade and Bruns, 1968). Later in 1998, Anderson discovered that caveolae is not just lipid raft with sphingolipids and cholesterol, but more of a platform regulating various signaling activities (Anderson, 1998).

Caveolin as a key component protein of caveolae and has shown to be a critical regulator in multiple cellular processes such as endocytosis, transcytosis, and cellular metabolism (Parton and Richards, 2003; Williams and Lisanti, 2004; Williams et al., 2005; Watanabe et al., 2009). As a protein family, caveolin has three members: caveolin-1, caveolin-2, and caveolin-3. Caveolin-1 and caveolin-2 are found in all types of cells, and caveolin-3 is mostly expressed in skeletal muscles (Liu et al., 2002; Gupta et al., 2014). Particularly, caveolin-1 has been widely investigated and was identified as the main protein in the caveolin family (Bastiani and Parton, 2010). Numerous studies have examined that caveolin-1 is closely associated with the development of various diseases, including pulmonary hypertension, vascular abnormalities, metabolic diseases, and cancer (Razani et al., 2001; Razani et al., 2002; Cohen et al., 2004; Cao et al., 2008; Austin et al., 2012). Recently, with the remarkable development of cancer research, extensive findings have indicated that caveolin-1 is a key factor in the occurrence, progression and prognosis of tumor (Witkiewicz et al., 2009a; Doherty and McMahon, 2009; Nassar et al., 2013; Chanvorachote et al., 2014; Li et al., 2018; Maiuthed et al., 2018). Firstly, abnormal expression of caveolin-1 has been found in several types of malignant tumors, such as gastric cancer, breast cancer, lung cancer, and prostate cancer (Sunaga et al., 2004; Witkiewicz et al., 2009b; Celus et al., 2017; Maiuthed et al., 2018; Lin et al., 2019). Furthermore, many studies demonstrated that caveolin-1 contributes significantly in the modulation of multiple cellular processes of cancer, including migration, metastasis, survival, and angiogenesis (Goetz et al., 2008; Ho et al., 2008; Tang et al., 2012; Faggi et al., 2015; Maiuthed et al., 2018; Yamao et al., 2019). Therefore, caveolin is believed to be a promising therapeutic target as well as an effective prognostic biomarker for cancer treatment.

2) The role of caveolin in carcinogenesis

Since aberrant expression of caveolin was found in several solid tumors, caveolin was considered to be a validated biomarker of cancer prognosis. However, the exact role of caveolin in tumorigenesis remains controversial (Martinez-Outschoorn et al., 2015). In some early studies, caveolin was initially regarded as a tumor suppressor. Insufficient caveolin-1 is able to induce carcinogenesis (Drab et al., 2001; Marx, 2001), and downregulation of caveolin-1 enhances tumor cell invasion and metastasis (Lu et al., 2003; Williams et al., 2004). In more recent studies, Du *et al.* suggested that complex formation of caveolin-1 in lipid transfer domain contributes tumor suppression (Du et al., 2012), Neofytou et al. reported similar results that the loss of caveolin-1 expression is significantly correlated with the poor prognosis of patients with colorectal cancer (Neofytou et al., 2017), Chatterjee et al. found that overexpression of caveolin enhances the sensitivity of tumor cells to paclitaxel and thus promotes apoptosis of tumor cells (Chatterjee et al., 2017), and Pavlides et al. indicated that stromal caveolin-1 may be a biomarker for human breast cancer such that a low expression of stromal caveolin-1 is associated with tumor recurrence, metastasis, and poor clinical outcome (Pavlides et al., 2009).

On the other hand, caveolin was found to be a tumor promotor. Lobos-González et al. discovered that high level of caveolin-1 expression promotes the invasion and metastasis of melanoma (Lobos-González et al., 2013), Seker et al. discovered that overexpression of caveolin reduced the survival time of patients with gastric cancer (Seker et al., 2017), and Huang et al. indicated that increased expression of caveolin-1 causes malignant transformation of glioma cells (Huang et al., 2018). Interestingly, in lung cancer but between different subtypes, Sunaga *et al.* found opposite effects of caveolin-1, such that caveolin-1 functions as a tumor suppressor in small cell lung cancer, but a tumor promoter in non-small cell lung cancer (Sunaga et al., 2004).

Although the role of caveolin in carcinogenesis remains to be elucidated, Carver and Schnitzer suggested that caveolin has the role of tumor suppressor mainly in the earlier stages of cancer, while in later stages of cancer, it supports the metastasis and survival of cancer cells due to the biological functions of caveolin in molecule delivery and signal modulation (Carver and Schnitzer, 2003). Therefore, this finding could possibly explain, at least in part, the contradictory results on the role of caveolin in tumorigenesis.

3) The potential caveolin-related targets in cancer therapies

Since caveolin influences multiple processes of tumor and the signal transduction of tumor cells, there are several potential caveolin-related targets in cancer therapies that could be further discussed.

Tumor microenvironment refers to the local stable-state environment which influences the occurrence and metastasis of tumor, and fibroblasts is one of the key element in tumor microenvironment (Shimizu et al., 2017; Yamao et al., 2019). Goetz et al. reported that microenvironment remodeling by caveolin-1 fibroblasts leads to the migration and invasion of cancer cells (Goetz Jacky et al., 2011), Yamao et al. found that downregulated caveolin-1 expression reduces the invasiveness of pancreatic cancer cells (Yamao et al., 2019), and Shimizu *et al.* discovered that caveolin-1 fibroblasts could induce the growth and metastasis of tumor cells in lung cancer (Shimizu et al., 2017).

Autophagy is a fundamental process for cytoprotection and intracellular homeostasis, and caveolin has been shown to be a critical regulator in autophagy of tumor cells with oxidative stress (Martinez-Outschoorn et al., 2010a; Martinez-Outschoorn et al., 2010b). Liu *et al.* indicated that caveolin-1 inhibits autophagy of cancer cells in hepatocellular carcinoma, providing potential target for autophagy inhibition as a novel treatment (Liu et al., 2016). More recent studies demonstrated that caveolin overexpression and phosphorylation are positively correlated with autophagy induction, which lead to tumor metastasis and cancer cell survival (Ortiz et al., 2016; Meng et al., 2017; Jiang et al., 2022). Evidence from Luanpitpong *et al.* revealed that oxidative stress induces the decomposition of tumor-related fibroblasts which further promotes tumor growth, and caveolin-1 has the capability to reverse this process (Luanpitpong et al., 2010). Sotgia et al. further suggested that oxidative stress is a key factor in the loss of stromal caveolin-1 via autophagy, and thus provided guidance for the application of antioxidants in cancer therapy (Sotgia et al., 2012). Another finding of Volonte et al. showed that caveolin-1 reduction inhibits oncogene-induced oxidative stress, thereby blocks the carcinogenesis pathway (Volonte et al., 2018).

In addition, some recent studies have shown that tumor cells with drug resistance exhibited an upregulated expression of caveolin. Wang et al. observed that silencing of caveolin-1 significantly reduces drug resistance of breast cancer cells (Wang et al., 2014), and Yuan et al. introduced the promoting effect of caveolin-1 in drug resistance of human gastric cancer cells (Yuan et al., 2013). Other clinical studies discovered the critical role of caveolin in the effectiveness and drug resistance of trastuzumab, providing promising targets and novel research directions for clinical applications (Pereira et al., 2018; Sung et al., 2018).

With the rapid development of multidiscipline, caveolin has been combined with novel techniques recently. Nanotechnology has been essentially involved in drug delivery and was benefited from endocytosis mediated by caveolin (dos Santos et al., 2011). Shamay et al. found that nanoparticles can selectively target kinase inhibitors in caveolin-dependent cancer models, and this finding would improve the computational modeling of nanomedicine designs (Shamay et al., 2018). Another study using photodynamic therapy reported that photosensitizers and caveolin-mediated endocytosis are critically involved in the precise targeting of cancer photodynamic therapy (Liu et al., 2022). Together, these studies provided promising targets and novel research directions for the therapeutics of cancer.

## Conclusion

In summary, our bibliometric analysis shows that caveolin continues to be of interest in the field of cancer research. The United States ranked first with scientific productivity and was closely cooperated with other countries. At the same time, Thomas Jefferson University from the United States have conducted in-depth research in this area. Moreover, Michael P. Lisanti was the most productive author in this domain. The research frontiers of caveolin has undergone a shift from the regulation of cancer signaling to potential targets for cancer therapy and novel techniques. As such, our study conducted a systematic bibliometric analysis of literature on this subject, and provided a data-based reference for the guidance of future investigation in this field.

## Limitations

The current study systematically visualized the relationship between caveolin and cancer. However, there are some limitations that should be mentioned as well. First, we only included publications that are written in English, thus potential findings published in other languages may not be covered. Second, only publications retrieved from the WOSCC database were selected, and documents from the Google Scholar database and the Scopus database were not searched. Therefore, some of the relevant studies may have been omitted. Third, we only obtained the published literature during 2003–2022, some of the very recent studies were thus not included in the analysis.

## Data Availability

The original contributions presented in the study are included in the article/supplementary material, further inquiries can be directed to the corresponding author.
